# Oxford graduates' perceptions of a global health master's degree: a case study

**DOI:** 10.1186/1478-4491-9-26

**Published:** 2011-10-21

**Authors:** Emma Plugge, Donald Cole

**Affiliations:** 1 Department of Public Health, University of Oxford, Old Road Campus, Old Road, Oxford OX3 7LF, Oxfordshire, United Kingdom of Great Britain and Northern Ireland; 2 Department of Public Health Sciences, University of Toronto, Toronto, ON, Canada

## Abstract

**Introduction:**

Low and middle-income countries suffer an ongoing deficit of trained public health workers, yet optimizing postgraduate education to best address these training needs remains a challenge. Much international public health education literature has focused on global capacity building and/or the description of innovative programmes, but less on quality and appropriateness.

**Case description:**

The MSc in Global Health Science at the University of Oxford is a relatively new, full-time one year master's degree in international public health. The programme is intended for individuals with significant evidence of commitment to health in low and middle income countries. The intake is small, with only about 25 students each year, but they are from diverse professional and geographical backgrounds. Given the diversity of their backgrounds, we wanted to determine the extent to which student background influenced their perceptions of the quality of their learning experience and their learning outcomes. We conducted virtual or face-to-face semi-structured individual interviews with students who had graduated from the course at least one year previously. Of the 2005 to 2007 intake years, 52 of 63 graduates (83%) were interviewed. We used thematic analysis to analyze the data, then linked results to student characteristics.

**Discussion:**

The findings from the evaluation suggested that all MSc GHS graduates who spoke with us, irrespective of background, appreciated the curriculum structure drawing on the strengths of a small, diverse student group, and the contribution the programme had made to their breadth of understanding and their careers. This evaluation also demonstrated the feasibility of an educational evaluation conducted several years after programme completion and when graduates were 'in the field'. This is important in ensuring international public health programmes are relevant to the day-to-day work of public health practitioners and researchers in low and middle-income countries.

**Conclusions:**

Feedback from students, when they had either resumed their positions 'in the field' or pursued further training, was useful in identifying valuable and positive aspects of the programme and also in identifying areas for further action and development by the programme's management and by individual teaching staff.

## Background

### The importance of public health training initiatives

The World Health Organisation (WHO) has highlighted the importance of public health in improving population health across the globe and the significant negative impact of the deficit of trained public health workers in low and middle-income countries [1]. Undoubtedly further development of public health education is a part of the solution to this problem, but exactly how, where and by whom this should be done is contested [2]. There is considerable debate over the role that postgraduate education in all countries has to play in addressing the training needs [3]. The majority of schools of public health are in high income countries rather than in those countries with the most significant deficit of skilled public health workers. Of course this raises questions of equity but also of the appropriateness of programmes for those who intend to work in low and middle income countries (LMICs). Exactly how well prepared are graduates to improve population health, especially that of the marginalized and socially excluded?

Thus the focus has shifted from not only the quantity of training that is provided but also the quality and appropriateness of that training [2]. These developments are mirrored in the published literature: to date, much of the literature on international public health education has focused on its role in global capacity building for public health and/or the reporting of innovative programmes [4-8]. However, there has been some published evaluation of educational initiatives [4,5,9]. Such a move also reflects the increasing emphasis on quality assurance and enhancement in high income countries, e.g. the Bologna process and resultant Tuning process in the developing European Higher Education Area [10].

### The experience of international students

The educational research examining student learning in higher education identifies a number of factors affecting student learning [11,12]. These not only include aspects of the course and the host department--'the learning and teaching context'--but also student features such as their prior expectations, their perceptions of the context and their approach to learning [12]. With the growing internationalization of higher education [13], educational researchers have turned to examinations of the experiences of students, the challenges for faculty, and the opportunities for institutions in a wide range of programmes, although primarily at the undergraduate level. Cross-cultural variation in learning styles, perceptions of student and teacher roles and course evaluations among 'overseas' versus domestic students have been explored [14]. Other authors have focused primarily on 'non-English speaking background students' and the challenges of supervising them in English-speaking programmes [15]. More recent work has approached 'international' students as an opportunity for programmes to examine their own weaknesses and to respond with innovative curricula, supporting diversity and benefitting all students, no matter what their origins [16]. We found little research specifically on the experience of international students in public health training programmes in high income countries despite the relevance and established values of their 'voices' in enhancing the educational experience [17].

## Case description

### The MSc in Global Health Science, University of Oxford

The MSc in Global Health Science at the University of Oxford is a relatively new, full-time one-year master's degree in international public health. It is based in the Department of Public Health but draws on the university's strengths in a wide range of relevant disciplines, including tropical and infectious medicine, vaccinology, health economics and development studies. Upon completion of the programme, students should be self-directed and original in tackling problems in global health and equipped to continue to advance their knowledge, understanding and skills further in research or professional practice in the field of global health. The programme is intended for individuals with significant evidence of commitment to health in low and middle income countries. The intake is small with only about 25 students accepted each year, but the students are from diverse professional and geographical backgrounds. In 2008-2009, the students came from seventeen different countries with two-thirds from low or middle income countries, and 50% were not mother-tongue English speakers.

Most teaching is conducted in small groups. Each module comprises 10 to 14 'sessions', of approximately three hours. The sessions include a didactic component followed by an appropriate group activity. For example, in the 'statistical concepts for global health' module, this activity may involve using a computer package to analyse data. The programme includes both compulsory modules and optional modules. Students study the four compulsory modules in the first term: challenges in global health, principles of epidemiology, statistical concepts for global health, and public health and health policy. In the second term, students select two modules from six options: health economics; international development; health, environment and development; maternal and child health; tropical medicine; and vaccinology. The breadth of modules, ranging from the biomedical approach of vaccinology to the social sciences orientation of international development, enables the multidisciplinary student body to pursue study of relevance to their professional interests.

The largely theoretical nature of the first two terms contrasts with the third term, in which students are placed at an approved site in the United Kingdom of Great Britain and Northern Ireland (U.K.) or overseas to apply their knowledge and deepen their understanding of global public health. The majority of students choose to go overseas to one of several approved placement sites. Several sites are part of the Tropical Medicine network (http://www.tropicalmedicine.ox.ac.uk/home). Students undertake an eight-week project which may be research or policy focused and which contributes to a 10 000- word dissertation, which they are required to submit as part of their final assessment (See Table 1).

**Table 1:** Key Components of the MSc in Global Health Science, University of Oxford

Timing	Components
Michaelmas Term October to mid-December	Students study all FOUR compulsory modules: Challenges in Global Health Public Health and Health Policy Principles of Epidemiology Statistical Concepts for Global Health

Hilary Term Early January to mid-March	Students study TWO modules from six options: Health Economics International Development Health, Environment & Development Maternal and Child Health Tropical Medicine Vaccinology

Trinity Term Late April to late June	Placement (U.K.-based or overseas)

Long Vacation Late June to mid August submission deadline	Write up of dissertation based on placement

### Assessing factors influencing student experience and quality

Quality assurance (QA) measures have been in place since the MSc started in 2005 and have been used to develop and improve the programme. Among potential methods to expand these measures is follow-up or 'tracking' of graduates; this has been used for QA of higher education programmes and in the educational evaluation literature, not only to update alumni data but also to gather graduates 'voices'. Given the diversity of both the professional and geographical background of the student body on the master's, we were particularly interested in the extent to which the varied background of students influenced their perceptions of their learning experience, including appropriateness, and their learning outcomes. This paper reports on this specific aspect of our work through the eyes of students themselves, and explores the implications of these findings for course organizers.

## Evaluation

We sought out graduates of the first three years of the course: 2005-6, 2006-7, and 2007-8. We devised a semi-structured interview guide which covered the student trajectory--from applying to the MSc until their current work or study activities--informed by the literature on international students and international public health training (Appendix 1). The course director and head of department sent a personal letter to each graduate via email, indicating the nature of the QA review. A sabbaticant with expertise in international public health education followed up with requests for an interview time via Skype (most interviewees being outside the United Kingdom), telephone (U.K., Europe and occasional hard to reach places, e.g. a Kenyan refugee camp), or in person (those working or studying in and around Oxford). The sabbaticant provided the interview outline but indicated that it would be adapted to respond to both the interests of interviewees and any significant issues that arose during interviews. During the interview, the sabbaticant reiterated the purpose and his role. He indicated that he would be typing notes during the conversation and that every effort would be made to assure anonymity of their responses, prior to obtaining verbal consent to continue.

The interviews were not tape recorded, rather the interviewer made detailed notes at the time. Respondent validation was conducted by checking key statements with the participant at the end of the interview. The detailed notes were uploaded into NVIVO 8. The data were analyzed using thematic analysis; and the two authors independently read, reread and categorised the data. They continually checked for the accuracy and consistency of interpretations by constant comparison, and searched for negative cases. The emergent themes--as identified independently by the two researchers--were compared, and any differences resolved by discussion. They also independently examined the data for the emergence of themes by both income status of country of origin and by professional background.

Countries were categorized according to the World Bank classification (low, middle and high income). Individuals were classified according to whether or not they were clinicians, that is a nurse, physician (or medical student) or allied health professional such as a nutritionist. All medical students had completed their first degree and were undertaking the MSc prior to completing their clinical training and qualifying as physicians. Physicians were further classified according to whether or not they were training in public health.

### Findings from the evaluation

The response was enthusiastic: 13/16, 17/23, and 22/24 by year, or 52/63 (82.5%) overall. Based on the World Bank per capita income country classification [18] over half the responding graduates came from high income countries (27/52); 15/52 from middle countries; and 10/52 from low income countries (see Table 2). Clinicians constituted less than half of the participating graduates, though by far the majority from MICs. They were primarily physicians and a few medical students, but included a nurse, nutritionist and dentist. Physician specialities ranged from general practitioners, through public health physicians in training or practice, to infectious disease and oncology specialists.

**Table 2:** Graduate participants of the Master in Global Health Sciences by profession and geographic origin

Country income category*	Profession		
	
	Clinician	Non-Clinician	Totals
High	7	20	27

Middle	13	2	15

Low	2	8	10

Totals	22	30	52

* as classified by the World Bank. Accessed 26 May 2010 at http://web.worldbank.org/WBSITE/EXTERNAL/DATASTATISTICS/0,,contentMDK:20420458~menuPK:64133156~pagePK:64133150~piPK:64133175~theSitePK:239419,00.html

All students, irrespective of background, appreciated the small class size, the diversity of students in the class, and the contribution their learning during the MSc had made to their careers. The quotes used below are representative of the majority of respondents except when we have highlighted the fact that it was a minority view.

### The value of a small, diverse group

The students felt that the small, diverse class facilitated their learning in a number of ways. The small size enabled the whole group to interact and promoted verbal exchange among all students. One student remarked that the master's had

"*more group work, so people could help each other.'*

Physician, lower income country, 2007-08

Another student emphasised the importance of keeping the class size small to ensure that all participated in class discussions. He stated,

'Another good thing is that the class is relatively small. Above a critical mass it is hard for everyone to contribute.'

Physician, middle income country 2005-06

Others felt the small group enabled the students to form good relationships, both within and beyond the formal teaching sessions, which facilitated peer learning. Given the diversity of the group in disciplinary, professional and cultural backgrounds, there was a great deal to be learned from the other students.

*'I really enjoyed the people who were part of the programme, the different health care and geographic backgrounds. The small class size was so conducive to forming good relationships. That level of diversity in a class of 20 or so was phenomenal: different backgrounds, five continents. It was a great experience from that standpoint*.

Non-clinician, high income country 2006-07

One student succinctly described the 'international mosaic of a class'. Most students felt very positive about the opportunities this 'mosaic' presented them for learning about global health.

'One of the best aspects was how students were recruited from not only diverse countries but diverse educational backgrounds. ... I learned at least as much from the way other students reacted to what we were taught. Most students had something to contribute of their experience.'

Non-clinician, high income country 2006-07

Despite a shared admiration for her fellow students, a non-medically trained student harboured preconceptions regarding the likely input from those who were medically qualified, which she learned were largely unfounded:

'The most amazing part of the programme was the people. The students that they put together for my year were phenomenal. [I] felt really inspired, awed by the level of expertise, from physicians in Sudan to Rhodes scholars. There was a wide variety of backgrounds and a lot of medical hard science people, but really open minded.'

Non-clinician, high income country 2006-07

### Concerns with diversity

A minority of students, all physicians from HICs, were less sanguine. One noted that the 'diversity of backgrounds is a challenge for the students as well as the course developers.' As a Rhodes scholar from a high income country herself, she explained:

'There were a lot of dominant personalities and this made group work difficult. More than half the Rhodes scholars were from the developed world and they dominated everything, took over from developing world students.'

Another student was concerned that some students were effectively unable to participate because they had an insufficient command of English. He said,

*'Some students' limited English competence slowed down discussions and limited [them]. Therefore English requirements need to be strict*.

Physician, high income country 2007-08

Another remarked on what he found to be a minor but irritating aspect of a diverse class:

'There were people with different cultural backgrounds, different experiences of organization, repeatedly arriving late for class.'

Physician, high income country 2005-06

He believed that his learning was being disrupted by this behaviour but could also recognize it might be quite acceptable in some cultures.

Disciplinary training backgrounds also posed challenges. Students were able to appreciate the challenges for course design posed by very different levels of knowledge and understanding of core concepts,

*'Such a diverse group, we were, with such varied levels of skills*.

Non-clinician, lower income country, 2005-06

'It is very difficult to design an epi and stats course that takes students with very different backgrounds. Some already knew as much as was going to be taught, others didn't feel comfortable with numbers, so [we] had reviews and refreshers in second term for those [who were] confused.'

Physician, high income country 2007-08

### Contribution to future careers

Another positive aspect of the programme, the contribution it made to career development, appeared to differ by disciplinary background, though not geographic origins. Differences emerged between non clinicians and clinicians, and also within the latter, depending on whether he/she was a physician clinician or undertaking public health specialty training. The clinicians were not exam oriented but rather talked in terms of the MSc broadening their horizons, enabling them to understand how their clinical work fitted into a much larger picture.

'I intend to work somewhere in East Africa and I want to work clinically, but also realise that many problems have to be approached from a public health perspective to be of any use. For example, we must address why children are getting diarrhoea as well as treat a child with diarrhoea.'

Medical student, high income country 2006-07

'The course provided a different perspective to the microscopic clinical focus, an overview. For example, in oncology, billions of dollars are spent on preventable cancers like liver cancer in South-East Asia caused by flukes. It's untreatable when [patients] present and but they can't afford earlier treatment. I can now see the public health view.'

Physician, middle income country 2005-06

This clinician went on to note that 'politics is such an important cause of disease across the world.' For him, the programme had opened his eyes to the wider determinants of health. Another clinician remarked that he had been very 'narrow minded' but that the MSc had 'helped him see the breadth, opened his mind'. A physician from a low income country described how the course had given him practical skills which enabled him to work more effectively as a district health services manager:

'The MSc greatly contributed to my work. It gave me a broader view of how to implement initiatives, of monitoring and evaluation and translating national policies at a district level. I became more aware of the global situation... I became more able to analyse things more critically so that the team thinks through what they are here for, understands the targets and the role of indicators... I know better to critique what donors may suggest, in light of both evidence/information, so that it better matches community needs.'

Physician, low income country 2007-08

In contrast, for physicians training in public health in the U.K., one of the main benefits of the programme was providing them with the necessary information and skills to prepare for their postgraduate exams ('Part A') before the U.K. Faculty of Public Health.

Those who were not physicians felt the MSc gave them time to explore their own interests and to decide how they wanted to work within public health thenceforth among a range of options:

'The MSc was helpful. It gave us the opportunity for one on one; we were able to ask all sorts of questions even those that you might of think as stupid... There was a good mixture of formal and informal teaching. It confirmed my desire to do doctoral studies and research.'

Clinician, non-physician, low income country 2005-06

'The master's led me to refugee health, nomadic populations. It is very hard to implement programmes in the refugee area. The programme pushed me into working in the field, something more applied. I could see how much I could learn in the field, how to work with UNHCR, etc. Although it shaped my interest in working with this kind of population, this kind of life, it did not prepare me for this kind of life. I had an academic understanding of refugee camps, but it's not what you see in reality.'

Non-clinician, high income country 2006-07

'I'm working in a very different capacity now; before [the MSc] I worked as staff or extra hands, now [I am] in a leadership capacity... they wanted someone with expertise in public health and youth with HIV, programme evaluation - I didn't even speak that language before doing the MSc... I think about it often as it was a really important year for me. When I first got back I thought that I wanted to have global health in the job [yet] my understanding of global health prepares me for so many things.'

Non-clinician, high income country 2005-06

## Discussion

The findings of this evaluation suggested that all MSc GHS graduates who spoke with us, irrespective of background, appreciated the curriculum structure, drawing on the strengths of a small, diverse student group and the contribution the programme had made to their breadth of understanding and their careers. We also demonstrated the feasibility of an educational evaluation drawing out students' voices--and conducted--several years after programme completion, when graduates were 'in the field'. Such evaluation is important in ensuring international public health programmes are relevant to the day-to-day work of public health practitioners and researchers in low and middle-income countries. Given the paucity of available research, our exploratory study is a contribution to the existing literature.

Study in small groups of less than 30 students has been advocated as a good educational method to facilitate interaction among students, not just with the instructor [19]. An effective group not only 'recognizes individual differences but actually exploits them' [19]. Our findings certainly suggest that the diversity of a student group promoted students' learning--many graduates eloquently described the extent of their learning from fellow students. However, educational research has also shown that potential problems can occur with group work; the teacher may dominate, one student may dominate, students may not prepare for sessions or they may simply want to be given the answer rather than discussing possible solutions [20]. On this MSc, the dominance of particular students, usually from high income countries, appeared to be a problem, although it was an issue mentioned by a minority of students. However, similar concerns had also been raised in other QA fora: both written feedback from individual students at the end of each week and verbal reports from the class representatives to the course committee. The Course Director should play a key role in ensuring that all teachers on the MSc find better ways to use and support diverse learners to enable the benefits to exceed the challenges associated with the course's small group design.

The geographic diversity of the group--i.e. the fact that they came from many different countries--appeared to be important to all students and was, on the whole, regarded as a very positive aspect of the programme. Such student endorsement of diversity emphasises the importance of recruiting students from all country income strata, benefiting learning and enriching university experiences, as has been emphasized by the more recent literature on international students [16]. Unfortunately many good students from low and middle income countries do not have access to enough funds to pay the university fees and living expenses in Oxford, particularly non-clinicians who might make important contributions to the public health workforce in informatics, surveillance, health promotion or policy roles. Hence, a key part of securing the future of both this MSc and other global health programmes, involves securing scholarships for students from low and middle income countries.

In this evaluation, the differences noted in programme contributions to graduate careers varied by professional group. Public health physicians' focus on learning to pass postgraduate exams is consistent with the educational literature on the adoption of strategic approaches to learning by medics [21]. Students demonstrating a strategic approach want to fulfil assessment criteria and so choose to use a surface or deep approach depending on what they feel will produce the most successful results [22,23]. These particular physicians were not only taking the MSc exams but also the U.K. Faculty of Public Health's higher professional exams. These findings suggest that the additional burden of the Faculty's assessment steered these students away from the deep learning the programme sets out in its aims. Nevertheless, the adoption of this learning approach by some did not seem to adversely impact on other students' learning, with some of the most outstanding statements on the master's contribution coming from non-clinicians, or those returning to LMICs in public health management roles.

Clinicians' enhanced understanding of the wider determinants of health and their greater breadth of knowledge have been highlighted in other educational evaluations of public health master's programmes which cite broadening of how clinicians' view 'disease'[4]. The very practical applications of learning by physicians from low income countries who had returned immediately after completing the MSc is also consistent with a strength cited among other public health master's programmes, which provide appropriate skills and knowledge that can be applied when at work [4,5,9].

This was a carefully conducted qualitative evaluation in which the researchers aimed to ensure good data quality in a number of ways in the planning and conduct of the study and in the data gathering and analysis. Our follow-up qualitative approach enabled a large proportion (over 80%) of graduates to share their voice. The broad range of perspectives captured in this way was supported by other written and verbal evaluation data elicited regularly from students whilst on the course. Nevertheless, the interviews were not tape recorded and respondent validation was timely but brief. Despite the interviewer's considerable experience of capturing data in detailed notes, some more nuanced themes may have been missed. However, this method was undoubtedly able to capture key issues such as the value of small diverse classes in enhancing learning. Furthermore the data analysis was carried out independently by both authors who searched for negative cases and checked the consistency and accuracy of interpretations and the application of codes by constant comparison.

The appropriateness of classifying students by the income strata of their country of origin poses problems. Such students may have studied and worked in a high income country for several years prior to studying for the master's and therefore their perceptions of the programme would be influenced by this prior experience. However, when we examined the data, only three students who were classified as coming from a middle or from a low income country had been in the U.K. or another high income country for more than one year prior to the master's.

## Conclusions

This evaluation provided valuable information on key aspects of the MSc programme: class size and the feasibility of evaluating the appropriateness of the programme curriculum when students have graduated and are pursuing careers in or related to public health. The findings suggest that all students, regardless of professional background, value small group work with a class from diverse cultures and disciplines, although difficulties were also highlighted by a minority of students. This has important implications for the programme's management in supporting teachers to develop effective ways of teaching diverse student groups.

The value of feedback from graduates when they have resumed their positions 'in the field' was very apparent. They provided valuable information on the useful and positive aspects of the programme but also identified areas for further action and development by teaching staff. Given the importance of the debate over the role that postgraduate education in all countries has to play in addressing the public health training needs of low and middle-income countries, our limited evaluation highlights the need for and feasibility of further educational evaluations which specifically examine the contribution public health programmes have made to the day-to-day work of public health practitioners and researchers in low and middle-income countries.

## Abbreviations

HICs: High income countries; LMICs: Low and middles income countries; QA: Quality Assurance; U.K.: United Kingdom of Great Britain and Northern Ireland; WHO: World Health Organisation

## Competing interests

EP is course director of the MSc Global Health Science at the University of Oxford.

DC declares that he has no competing interests.

## Authors' contributions

EP and DC designed the study. DC collected the data and, together with EP, analysed and interpreted the data. EP wrote the first draft of the paper and DC critically reviewed this and contributed substantially to all redrafts. Both EP and DC read and approved the final manuscript.

## Appendix 1

### Graduate interview guide

A. What background did you bring to the MSc GHS? [probes: education, professional experience, approach to learning, other]

B. What lead you to Oxford? And what were your expectations?

C. What was your overall impression of the MSc GHS? What aspects of the MSc GHS programme were most helpful/useful? [probes: research placement, dissertation, modules, personal tutor, core staff, other]

D. Were there aspects of the MSc GH programme which were less helpful/useful? [probes: research placement, dissertation, modules, personal tutor, core staff, other]

E. What have you done since graduation?

F. How does the MSc GHS contribute to your current work? [prompt: did it help with you obtaining current position?]

## References

[B1] World Health Report 2006 Working Together for Health 2006 Geneva: World Health Organization

[B2] Petrakova A Sadana R Problems and progress in public health education Bull World Health Organ 2007 85 12 963 5 10.2471/BLT.07.046110 18278257 PMC2636301

[B3] Sadana R Petrakova A Shaping public health education around the world to address health challenges in the coming decades Bull World Health Organ 2007 85 12 902 10.2471/BLT.07.049247 18278241 PMC2636291

[B4] Alexander L Igumbor E Sanders D Building capacity without disrupting health services: public health education for Africa through distance learning Human Resources for Health 2009 7 1 28. 10.1186/1478-4491-7-28 19338669 PMC2678972

[B5] Le L Bui Q Nguyen H Rotem A Alumni survey of Masters of Public Health (MPH) training at the Hanoi School of Public Health Human Resources for Health 2007 5 1 24 10.1186/1478-4491-5-24 17949491 PMC2186353

[B6] Lopez A Caceres V Central America Field Epidemiology Training Program (CA FETP): a pathway to sustainable public health capacity development Human Resources for Health 2008 6 1 27. 10.1186/1478-4491-6-27 19087253 PMC2636840

[B7] Parkes MW Spiegel J Breilh J Cabarcas F Huish R Yassi A Promoting the health of marginalized populations in Ecuador through international collaboration and educational innovations Bull World Health Organ 2009 87 312 9 10.2471/BLT.07.045393 PMC2672579 19551240

[B8] Obbadi M The course of specialization in public health in Rio de Janeiro, Brazil, from 1926 to 2006: lessons and challenges Hum Resour Health 2010 8 4 10.1186/1478-4491-8-4 20205733 PMC2845089

[B9] Mokwena K Mokgatle-Nthabu M Madiba S Lewis H Ntuli-Ngcobo B Training of public health workforce at the national school of public health: meeting Africa's need Bulletin of the World Health Organization 2007 85 12 949 54 10.2471/BLT.07.044552 18278255 PMC2636306

[B10] Confederation of EU Rectors' Conferences and the Association of European Universities The Bologna Declaration on the European space for higher education: an explanation 2009 ec europa eu/education/policies/educ/bologna/bologna pdf

[B11] Ramsden P Ramsden P Learning from the student's perspective Learning to Teach in Higher Education 2003 2 London & New York: RoutledgeFalmer 209 32

[B12] Prosser M Trigwell K A model for understanding learning and teaching in higher education Understanding Learning and Teaching 1999 Buckingham: SRHE & Open University Press 10 25

[B13] Ninnes P Hellsten M Internationalizing Higher Education: Critical explorations of pedagogy and policy 2005 Hong Kong: Comparative Education Research Centre and Springer

[B14] Todd E McNamara D, Harris R Supervising overseas students: problem or opportunity? Overseas Students in Higher Education 1997

[B15] Ryan Y Zuber-Skerritt O Supervising post-graduates from non-English speaking backgrounds 1999 Buckingham, U.K. and Philadelphia, USA: Society for Research in Higher Education and Open University Press

[B16] Ryan J Carroll J Ryan J, Carroll J 'Canaries in the coalmine': international students in Western universities 2005 Teaching International Students.Abingdon,U.K. and New York,USA: Routledge

[B17] Jones E Caruana V Jones E Nurturing the Global Graduate for the twenty first century: learning from the student voice of internationalisation 2009 Internationalisation and the student voice: higher education perspectives. Routledge

[B18] World Bank Country classifications http://data.worldbank.org/about/country-classifications

[B19] Abercrombie J Nias J Teaching groups in higher education: types, purposes and techniques The Human Nature of Learning: selections from the work of M.L.J. Abercrombie 1993 Buckingham: SHRE and Open University Press

[B20] Ramsden P Teaching strategies for effective learning Learning to Teach in Higher Education 2003 2 London: RoutledgeFalmer 145 75

[B21] Newble DI Gordon MI The learning style of medical students Med Educ 1985 19 3 8 10.1111/j.1365-2923.1985.tb01132.x 3969021

[B22] Newble DI Entwistle N Learning styles and approaches: implications for medical education Med Educ 1986 20 162 75 10.1111/j.1365-2923.1986.tb01163.x 3724571

[B23] Newble DI Clarke RM The approaches to learning of students in a traditional and an innovative problem-based medical school Med Educ 1986 20 267 73 10.1111/j.1365-2923.1986.tb01365.x 3747871

